# Pharmacokinetic Pattern of Menbutone in Calves after Single Intravenous and Intramuscular Administration

**DOI:** 10.3390/ani14172540

**Published:** 2024-08-31

**Authors:** Raquel Diez, Jose M. Rodriguez, Cristina Lopez, Raul de la Puente, Matilde Sierra, M. Jose Diez, Nelida Fernandez, Juan J. Garcia, Ana M. Sahagun

**Affiliations:** Pharmacology, Department of Biomedical Sciences, Veterinary Faculty, Institute of Biomedicine (IBIOMED), University of Leon, 24071 Leon, Spain; rdielz@unileon.es (R.D.); jmrodl@unileon.es (J.M.R.); rpueg@unileon.es (R.d.l.P.); msiev@unileon.es (M.S.); mjdiel@unileon.es (M.J.D.); mnferm@unileon.es (N.F.); jjgarv@unileon.es (J.J.G.); amsahp@unileon.es (A.M.S.)

**Keywords:** choleretic, intramuscular, intravenous, menbutone, pharmacokinetics, calves

## Abstract

**Simple Summary:**

Menbutone is a drug that increases hepato-digestive secretions and has been used for more than 50 years in Europe to treat a wide number of digestive disorders in livestock and dogs. However, despite this use, little is known about its pharmacokinetics, more specifically in cattle. This work contributes to a better knowledge of the pharmacokinetics of menbutone when administered intravenously and intramuscularly to cattle (12 Holstein calves), being the first study to describe it in this animal species. After intravenous administration, the drug showed a moderate volume of distribution and a fast elimination from the body. After intramuscular administration, menbutone exhibited quick and high absorption.

**Abstract:**

Menbutone is a choleretic agent currently used in Europe to treat digestive disorders in livestock and dogs. Pharmacokinetic parameters were established in 4-month Holstein calves after intravenous (IV) and intramuscular (IM) administration. The drug was administered to 12 animals (10 mg/kg) for both IV and IM routes following a crossover design. Plasma samples were collected at various time points over 24 h and analyzed by reverse-phase high-performance liquid chromatography with a photodiode-array detector, following a method validated according to European Medicines Agency guidelines. Pharmacokinetic parameters were calculated using compartmental and non-compartmental methods. Menbutone followed a two-compartment open model after IV injection, with a total clearance (Cl) of 71.9 ± 13.5 mL/h/kg, an elimination half-life (t_½β_) of 4.53 ± 2.45 h, and a volume of distribution at steady-state (V_ss_) of 310.4 ± 106.4 mL/kg. Non-compartmental elimination half-life (t_½λ_) was 4.2 ± 1.1 h. After IM administration, drug pharmacokinetics was best described by a one-compartment open model. The peak plasma concentration (C_max_) was 15.1 ± 4.3 µg/mL; the time to reach C_max_ (t_max_), 1.66 ± 0.55 h; and the mean absorption time (MAT), 2.50 ± 1.42 h. Absorption was high, with a fraction of the dose absorbed (F) of 83.5 ± 22.4%. Menbutone was rapidly eliminated from plasma for both routes of administration, with a fast and high IM bioavailability.

## 1. Introduction

Gastrointestinal disorders account for a significant proportion of cases confronted in veterinary practice. In addition, certain digestive dysfunctions such as anorexia, constipation, or indigestion tend to be common as concomitant conditions in many animals. Menbutone, also known as genabilic acid, is an oxybutiric acid derivative. This drug has choleretic properties and has been used for several decades to treat digestive upsets (loss of appetite, indigestion, toxemia, or hepatic and pancreatic insufficiencies) in a variety of animal species, including different farm animals (cattle, sheep, goats, pigs), as well as in dogs. This drug improves the function of the gastrointestinal tract and stimulates all associated secretory processes, more specifically, bile, peptic, and pancreatic secretions. Despite this use, evidence for its clinical efficacy in any animal species is poor. In this regard, its choleretic effect has been described in healthy cattle [1]. The work of Lund and Lassen [2] indicated that the stimulating effect on bile and pancreatic juice lasted for 2–3 h in anesthetized animals, but no reference was provided to support this claim. Menbutone has also been described in a case report involving cattle as an additional treatment for monensin poisoning [3], but no evidence was presented about its efficacy.

The drug is administered parenterally (by intravenous (IV) or intramuscular (IM) routes) up to a dose of 10 mg/kg. In the European Union (EU) several veterinary medicines containing menbutone are currently available for use in most countries [4,5]. In the case of food-producing animals, the EU has considered that it is not necessary to establish a maximum residue limit (MRL) for this drug [6,7], which is an additional advantage for its use in livestock.

As happens with other choleretic drugs, and although the pharmacological action of menbutone has been described [1,2], its pharmacokinetics has hardly been studied, and sparse data are available in the Summary of Product Characteristics (SPC) of those veterinary medicinal products in which this drug is present. In a previous paper [8], the menbutone pharmacokinetic profile in sheep was fully described after both IV and IM administration, but it is the only domestic animal in which such a study has been carried out for this compound. An initial study of distribution and elimination was assessed in rats, but no pharmacokinetic parameter was given [2]. In sheep [8], the drug showed a fast elimination from the body after IV administration (t_½λ_ of 6.08 ± 2.48 h), reaching a C_max_ of 18.8 µg/mL at 3.75 h for the IM route. Regarding cattle, and despite its use in this animal species, to date, no studies have evaluated its pharmacokinetic properties. After IV administration, a concentration of 20 µg/mL has been measured at 1 h, diminishing to less than 1 µg/mL 8 h post-administration [6].

Rational use of drugs requires basic knowledge of pharmacokinetics in the target species to optimize clinical efficacy for the species and condition treated. Considering the current lack of information in cattle on the pharmacokinetic properties of this compound, and given its use in this animal species, the objective of this study was to determine the plasma pharmacokinetic pattern of menbutone in healthy calves after a single intravenous and intramuscular administration.

## 2. Materials and Methods

### 2.1. Drugs and Chemicals

A commercially available formulation (Digestosyva^®^ 100 mg/mL solution for injection, Laboratorios Syva S.A.U., Leon, Spain) containing menbutone was used for IV and IM administrations to animals. Menbutone reference standard (purity 98.99%) was procured from LGC-Dr. Ehrenstorfer (LGC Labor GmBH, Ausburg, Germany). The internal standard (IS), sparfloxacin, was obtained from Sigma-Aldrich (Merck, Darmstadt, Germany) (purity 98%). Acetonitrile (HiPerSolv Chromanorm), monopotassium phosphate (AnalaR Normapur), and acetic acid (HiperSolv Chromanorm) were purchased from VWR (Radnor, PA, USA), whereas methanol (LiChrosolv) and sodium hydroxide 1N were obtained from Merck (Madrid, Spain) and Panreac Quimica S.A. (Barcelona, Spain), respectively. HPLC-grade water was produced by a Millipore Milli-Q Gradient water purification system (Waters Corporation, Mildford, MA, USA). Oasis HLB 1cc 30 mg cartridges (Waters Corporation, Mildford, MA, USA) were employed for the solid phase extraction (SPE). 

### 2.2. Animals

The study was performed in compliance with the European and Spanish regulations for animal experiments [9,10]. All procedures were reviewed and approved in advance by the Institutional Animal Care and Use Committee at the University of Leon (protocol number OEBA-ULE-015-2023). A group of twelve clinically healthy female Holstein ruminant calves, 4 months old and weighing 95.4 ± 21.0 kg, were used. The study was conducted under field conditions at a Spanish commercial farm located in the province of León. Calves were isolated from other animals of the same age not included in this trial 7 days before the experiment for an acclimation period and maintained in these conditions until the end of the trial in a pen bedded with straw and wood shavings. None of them had been medicated within the month prior to the study, and they were deemed healthy based on physical examination developed by a veterinarian before starting the assay. Their diet consisted of a growing feed twice a day (daily ration 3–4 kg) with hay and straw *ad libitum* and free access to water. The growing feed was elaborated in a local feed mill from a formulation developed by a nutritionist and contained 16.5% crude protein, 4.3% crude oils and fats, 4.1% crude fiber, 4.5% crude ash, and a vitamin-mineral corrector. A veterinarian closely monitored the animals’ health throughout the experimental period. 

### 2.3. Design, Drug Administration and Sampling

A randomized two-period crossover design was planned. Animals were randomly assigned into one of the two treatment groups (*n* = 6 each) differing by the order in the route of administration: group 1: first IV and then IM, and group 2: first IM and then IV. Calves were weighed the day prior to each drug administration. To minimize the possibility of carryover effects and ensure the complete elimination of the drug from plasma, the second treatment was preceded by a 14-day washout period, and the routes of administration were switched for both groups. A commercially available formulation containing 100 mg/mL of menbutone was always used (Digestosyva^®^ 100 mg/mL solution for injection). The drug was administered, according to the SPC of the commercial formulation, at a single dose of 10 mg/kg for both routes of administration. For the IV route, a catheter (Vasocan 18G, Braun VetCare^®^ SA, Barcelona, Spain) was inserted into the left jugular vein just before administration and removed once dosing was completed. IV administration was made slowly, within 50–70 s. As for the IM administration, the drug was injected into the neck.

Blood samples (6 mL) were obtained via venipuncture from both jugular veins (alternating between left and right) into sodium heparin blood collection tubes (Vacutainer^®^, BD, Plymouth, UK). Sample collecting occurred at time 0 (before dosing), and at 0.33, 0.66, 1, 1.5, 2, 3, 4, 6, 8, 10, 12, and 24 h post-administration for the IV route, and at 0 (before dosing); 0.5; 1; 1.5; 2, 3, 4, 6, 8, 10, 12 and 24 h for the IM administration. For both IV and IM administrations, the first blood sample was collected from the side contralateral to the other one used for drug administration. Blood samples were immediately centrifuged for 20 min at 1500 rpm, and plasma was frozen at −20 °C until drug concentration analysis. 

### 2.4. Analytical Procedure

The study was conducted at the laboratory LAFARLE (University of Leon, Spain), which operates according to the Good Laboratory Practice regulations and is certified by the Spanish Agency of Medicines and Medical Devices. Menbutone concentrations were measured in plasma samples by high-performance liquid chromatography (HPLC) according to a previously published method [11]. Briefly, an internal standard (IS) (sparfloxacin, 20 µg/mL) was added to the plasma samples, and menbutone and IS were extracted from plasma by SPE with Oasis HLB 1 cc 30 mg cartridges (Waters Corporation, Mildford, MA, USA). Before performing SPE, the samples were deproteinized with 1 mL 10% acetic acid, shaken for 1 min, and centrifuged at 3000 rpm for 10 min. The supernatant was transferred into an SPE cartridge, washed twice with 1 mL HPLC grade water, and eluted with 1 mL mobile phase. 20 µL of the eluate was then injected into the HPLC system (Waters Alliance e2695, Waters Corporation, Mildford, MA, USA) equipped with photodiode array detector (PDA model 2998, Waters Corporation, Mildford, MA, USA). The optimal wavelength for quantification was set at 236 nm (menbutone) and 297 nm (IS). All procedures were performed at room temperature and not later than 6–8 weeks after sample collection.

### 2.5. Validation of the Analytical Method

The method used to determine menbutone concentrations was validated in cattle plasma in terms of selectivity, carryover, matrix effect, linearity, lower limit of quantification (LLOQ), accuracy, precision, and stability according to the EMA guidelines [12]. 

The selectivity of the bioanalytical method was evaluated by using six cattle blank samples for interference. Calibration curves (*n* = 3) were built using cattle blank plasma samples spiked at seven different concentrations of menbutone (0.2 to 100 μg/mL) and IS. The linear regression analysis was performed on known concentrations of menbutone against the ratio of area of menbutone/IS, and linearity was established by calculating the coefficient of determination (R^2^). LLOQ was defined with six blank samples spiked at the lowest concentration level (0.2 µg/mL). Within-run and between-run precision and accuracy were assessed by analyzing quality control (QC) samples at four concentrations (QC1 = 0.2; QC2 = 0.6; QC3 = 30 and QC4 = 75 µg/mL) in quintuplicate. Precision was calculated and expressed as the percentage of coefficient of variation (CV), and accuracy was reported as a nominal concentration of analyte and expressed as a percentage. Carryover was assessed by injecting a blank sample and a mobile phase after the highest concentration calibration sample and QC4. The matrix effect was evaluated with six different blank plasma samples spiked with menbutone and IS at two concentrations (QC2 and QC4). The stability of the analyte and IS in working solutions was also studied at two concentration levels (QC2 and QC4) under different storage conditions: at room temperature for 24 h; 4 °C for 24 and 48 h; and −20 °C for 7, 15 and 30 days. A complete freeze-thaw cycle was carried out every 3 days.

### 2.6. Pharmacokinetic Analysis

Compartmental and non-compartmental pharmacokinetic analysis of menbutone in plasma was performed for each animal individually using commercially available software (Phoenix WinNonlin, version 8.4; Certara Inc., St. Louis, MO, USA). Regarding compartmental analysis, one- and two-compartment open models with 1/y weighting were assayed. The best fit was determined based on the Akaike information criterion, comparing the residuals and the coefficients of variation (CV%) [13,14,15,16]. Pharmacokinetic parameters were calculated by standard methods [13,14].

Non-compartmental analysis was performed from the raw data, with expressions based on statistical moments theory [17] and standard formulae [13,14]. Plasma elimination rate constant (λ) was calculated by least squares regression of the logarithm of plasma concentration-time curve over the terminal elimination phase, and the elimination half-life (t_1/2λ_) as 0.693/λ. The area under the curve (AUC) and AUMC were calculated by the linear trapezoidal rule to the final concentration-time point, and extrapolated to infinity (dividing the final experimental concentration by the terminal slope). From these values, mean residence time (MRT = AUMC/AUC) and mean absorption time (MAT = MRT_IM_ − MRT_IV_) were determined. Maximum plasma concentration (C_max_) and the time to reach it (t_max_) were obtained directly by inspection of the data. 

The fraction of dose absorbed (F) was calculated using the equation

$$F=\frac{{AUC}_{IM}}{{AUC}_{IV}}\cdot 100$$

where AUC_IV_ and AUC_IM_ is the area under the curve after IV and IM administration, respectively.

### 2.7. Statistical Analysis

Pharmacokinetic parameters were expressed as mean ± standard deviation (SD). Statistical analyses were conducted using the IBM SPSS for Windows software package v. 26 (IBM Corporation, Armonk, NY, USA). Differences between compartmental and non-compartmental parameters were assessed in both routes of administration (IV and IM). In the case of non-compartmental parameters, differences between those parameters common to both IV and IM routes were also evaluated. Shapiro-Wilk test was used to test for normality. If normal, data were then compared using the paired *t* test; if not, the Wilcoxon signed-rank test was applied. Furthermore, a two-way ANOVA was also conducted to estimate the impact of the crossover design. In all cases, a value of *p* ≤ 0.05 was considered significant.

## 3. Results

### 3.1. Validation of the Method

As explained before, the quantitative HPLC method was validated for cattle plasma according to the EMA guidelines [12]. No peaks interfering with menbutone and IS were observed, which demonstrated the selectivity of the method. Standard curves were linear over the assayed range and showed optimal linearity, with *R*^2^ ≥ 0.996 (Table S1 details the curves calculated; see Supplementary File). LOD and LLOQ were 0.09 and 0.2 μg/mL, respectively, and the mean extraction recovery of the drug was 98.3 ± 8.9%. No carryover was detected after the analysis of samples. The intra- and inter-day precision range was calculated to be 5.8–9.2%, and intra- and inter-day accuracy was 99.0–107.4%. Precision and accuracy did not exceed 15% of the nominal concentration. Finally, the stability of menbutone and IS in plasma after storage at different times and conditions was always within the acceptance criteria (<15% of the nominal values) in all tests performed (accuracy 87–95%).

### 3.2. Pharmacokinetic Results

After IV administration, adverse effects were observed only in one of the animals (no. 9 (crossover period 1) in Figure S1, see Supplementary File), with transient dyspnea and lacrimation (less than 1 min). This animal fell down, although it recovered immediately. In the case of the IM administration, three animals exhibited momentarily signs of pain reaction (neck stretching and rubbing against objects) at the injection site (no longer than 3 min) (nos. 3 (crossover period 1), 9 and 12 (crossover period 2) in Figure S1, see Supplementary File). No other side effects were seen in any of the animals during the experimental trial.

**Figure 1:**
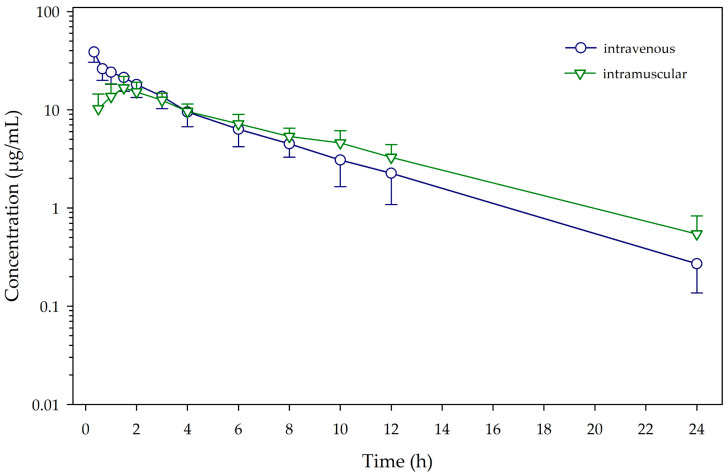
Plasma concentrations (mean ± SD) of menbutone obtained after intravenous and intramuscular administration (10 mg/kg) to 12 calves.

Mean ± SD plasma concentrations of menbutone versus time curves following IV and IM administrations are plotted in Figure 1. Individual concentrations for each animal are also presented as Supplementary Material (Tables S2 and S3, and Figure S1). Menbutone was quantifiable in plasma for the whole sampling period (24 h) in both IV and IM routes.

After IV administration, plasma menbutone concentration-time curves best fitted a two-compartment open model. Table 1 reports the main compartmental and non-compartmental pharmacokinetic parameters for this route of administration. Plasma concentrations declined rapidly, with short half-lives associated with the α-phase (t_1/2α_) and the terminal β-phase (t_1/2β_). Menbutone showed a high total body clearance (Cl) and a moderate volume of distribution at steady-state (V_ss_). Distribution was slightly higher in the central compartment (V_1_) than in the peripheral one (V_2_). As for non-compartmental parameters, the terminal half-life (t_1/2λ_) was quite similar to the mean residence time (MRT_0–∞_). Only non-compartmental V_ss_ was significantly higher than that calculated by compartmental analysis (paired *t* test).

Following IM administration, pharmacokinetic parameters were also calculated using both compartmental and non-compartmental methods (Table 2). For this route of administration, the pharmacokinetics of menbutone was best described as a one-compartment model with first-order absorption. A C_max_ of 15.1 µg/mL was reached at just over an hour and a half (t_max_ = 1.66 h). As for the AUC_0–∞_, the mean value was somewhat lower than that calculated for the IV route. A high IM bioavailability was achieved (F = 83.5%). Significant differences were found between IM compartmental and non-compartmental parameters for AUC_0–∞_ and Cl/F (paired *t* test) and C_max_ (Wilcoxon signed-rank test). When non-compartmental parameters from both routes of administration (IV and IM) were compared, significant differences were observed for AUMC_0–∞_, MRT_last,_ and MRT_0–∞_ (paired *t* test). Moreover, the statistical analysis revealed no significant differences between the two crossover trials that were carried out.

## 4. Discussion

Understanding the pharmacokinetics of a drug in the target species may help to ensure the appropriate use of medicines. This is the first report describing the pharmacokinetic pattern of menbutone in cattle following a single IV and IM administration. The results obtained illustrate a moderate distribution and a fast elimination of this choleretic drug after IV administration, as well as a fast and high IM absorption. 

With the exception of the approach recently carried out in sheep [8], no data are available for any other domestic animal species. A lack of pharmacokinetic information on this drug has been identified in cattle, an animal species of great importance in veterinary medicine. Therefore, we can only compare our results with those described in sheep, in which parameters were defined by non-compartmental methods after having administered the drug with the same commercial product and at the same dose (10 mg/kg) [8]. 

It should be noted that medicines containing this choleretic compound may be used in clinical practice by both routes of administration (IV and IM). Because of that, the knowledge of IV parameters is important from a therapeutic point of view and not only as the reference route of administration to then establish IM parameters. In the current study and after IV injection, menbutone plasma concentrations declined in a bi-exponential way, being rapidly eliminated from the body. For this animal species, we have found only a report where a concentration of 20 µg/mL was achieved in cows one hour after IV administration (10 mg/kg), with plasma values below 1 µg/mL after 8 h [6], values which were lower than ours. Although no further information is provided in that report, these disparities could be due to differences in the characteristics of the animals (gender, breed, age) and, above all, in the method used for the analytical determination of menbutone.

In our study, menbutone distribution was primarily limited to extracellular space, as indicated by V_ss_, and was nearly similar in both compartments or somewhat higher within the central one. For V_ss_, significant differences were revealed when comparing both compartmental and non-compartmental values. Thus, although a good fit was achieved after compartmental analysis, the non-compartmental value of this parameter should be used in comparisons. With regard to sheep [8], the volume of distribution was higher in cattle (V_ss_ = 259.6 mL/kg in sheep), and a faster clearance (Cl) was also noted (63.6 mL/kg/h in sheep). Together with this high clearance, a short t_1/2k10_ of 1.62 ± 1.15 h has been calculated in calves. Plasma t_1/2λ_ was approximately two-thirds of that obtained in sheep (6.08 h), whereas the area under the curve (AUC_0–∞_) was also lower than in the ovine species (165.0 µg·h/mL) [8]. Finally, MRT_0–∞_ values were similar to those calculated in sheep (4.23 h) [8]. These results show a lower exposure to menbutone in bovine species and correlated well with the rapid elimination described in both rats and sheep after IV administration [2,8]. 

As described in Section 2, a catheter was used to facilitate IV administration, although samples were obtained by venipuncture. The use of a catheter for both administration and blood sampling was initially considered, and a preliminary study was conducted to evaluate this option. However, the animals had to be significantly immobilized to fix the catheter properly (causing stress or damage to them). The usual behavior of calves (young, restless animals), which interact by licking, would make them try to remove the catheter if it disturbed them. Furthermore, an anticoagulant solution would be required to prevent the catheter from closing. All these aspects led to the decision to use the catheter only for IV administration and to obtain samples by venipuncture. It should also be noted that venipuncture was always performed by veterinarians with extensive experience in this technique, which minimized any impact on the welfare of the animals. On the other hand, with regard to the animal in which adverse effects were observed after IV administration, these are described in the SPC of the veterinary medicinal product administered and are likely related to a relatively rapid IV administration (it also showed the highest concentration at the first sampling point).

Regarding the IM route, data were best fitted by a one-compartment model. Although graphically data seemed to follow a two-compartment open model, the coefficients of variation obtained after having fitted data to this open model were too high [16], and it was necessary to discard the two-compartment open model and select the one-compartment model. For this route of administration and with the same dose (10 mg/kg), C_max_ was lower in calves than in ewes (18.8 µg/mL) [8], irrespective of whether compared with compartmental or non-compartmental parameter calculated in cattle. A shorter t_max_ was also obtained in calves, being nearly half the time as in ewes (3.75 h) [8]. This t_max_ value is also consistent with that calculated for MAT, which is also lower in cattle than in sheep (3.31 h) [8]. As happened in sheep, the menbutone absorption process was also faster than elimination. Regarding the fraction of dose absorbed, it was high in cattle but not complete as in ewes [8].

Regarding those factors that may explain the differences observed between the results of the current study and that carried out in sheep [8], as the same dose (10 mg/kg) and commercial formulation were used in both assays, the discrepancies found between both ruminant species may be attributed to other factors, such as differences in time sampling, specific-species differences or the age of the animals. In the current study, calves were already ruminant, although they were young animals (4 months), whereas sheep were adult ones (4–5 years). In veterinary medicine, data regarding the effects of age-related physiological changes on drug pharmacokinetics are limited. In this sense, cattle undergo major changes in composition and body structure as they mature into adult ruminants, which may affect pharmacokinetic processes [18]. Following IV administration, clearance of flunixin was slower in 2-month calves than at 8 months of age [19], and Cl/F and V/F were significantly lower in 3-week-old calves than in 6-month-old animals after subcutaneous administration of tulathromycin [20]. Thus, further studies should be conducted to evaluate the potential pharmacokinetic differences of menbutone among individuals of different age groups. 

In the case of the IM administration, they may also be related to differences in the site of injection, as in calves, menbutone was administered into the neck, and in sheep, administration was made into the gluteal muscle. Some studies have described the effect of this latter factor. It should be noted that the injection site in a certain animal species is related to the use and the economic value of this animal species, as well as the products obtained from it. For this reason, cattle are usually injected IM into the neck and sheep into the hind limb. In sheep, the neck area was considered moderately advantageous as an injection site compared to the thigh (semitendinosus muscle) for amoxicillin, mainly in terms of rate rather than extent of absorption [21], and something similar was described in horses with procaine penicillin G [22]. A large inter-individual variability has also been observed in plasma concentrations and pharmacokinetic parameters. Enterohepatic circulation described previously [2,6] may have also contributed to this variability. On the other hand, although a good fit was achieved, it cannot be excluded that this variability may have been influenced by having only 3 datapoints in the first hour after IV administration. 

Regarding the pharmacodynamics of this choleretic drug, it is difficult to predict the pharmacodynamic effect of menbutone plasma concentrations obtained in this study after IV or IM administration, as this relationship has not been established. The few existing studies on its pharmacodynamics show that menbutone at a dose of 10 mg/kg would be effective in achieving its choleretic action and that this effect occurred in the first hours after administration. In rats, one dose of menbutone had a choleretic effect of 2–3 h duration [2], and when the drug was given IV to steers at the same dose (10 mg/kg), a 4.5-fold increase in bile flow was described at least up to 6 h [1]. On the other hand, the present study is a single-dose one. Nevertheless, as indicated in the SPC of those veterinary medicinal products containing menbutone, a second dose may be clinically used after 24 h if necessary, the effect of which has not yet been evaluated.

## 5. Conclusions

This study generated novel information about menbutone pharmacokinetics. When administered either intravenously or intramuscularly to calves, elimination of the drug seems to be rapid. After IV injection, menbutone plasma concentrations best fitted a two-compartment open model and showed a fast elimination from the body. Plasma clearance appears to be faster than that reported in sheep. When the drug was administered intramuscularly, data were best described by a one-compartment open model and exhibited a high level of absorption. Additional studies are necessary to compare the pharmacokinetics of menbutone in calves versus adult cattle and establish if there would be relevant differences due to age.

## Figures and Tables

**Table 1:** Pharmacokinetic parameters (mean ± SD) of menbutone obtained after IV administration (10 mg/kg) to 12 calves.

Parameters	Two-Compartmental	Non-Compartmental
A (µg/mL)	171.7 ± 228.4	
B (µg/mL)	17.5 ± 9.1	
α (h^−1^)	3.71 ± 4.49	
β (h^−1^)	0.18 ± 0.07	
λ (h^−1^)		0.18 ± 0.04
k_10_ (h^−1^)	1.31 ± 1.52	
k_12_ (h^−1^)	2.11 ± 2.86	
k_21_ (h^−1^)	0.47 ± 0.28	
t_1/2α_ (h)	0.88 ± 0.76	
t_1/2β_ (h)	4.53 ± 2.45	
t_1/2λ_ (h)		4.17 ± 1.06
t_1/2k10_ (h)	1.62 ± 1.15	
V_ss_ (mL/kg)	310.4 ± 106.4	335.9 ± 70.0 ^a^
V_1_ (mL/kg)	165.4 ± 116.1	
V_2_ (mL/kg)	145.0 ± 78.2	
V_z_ (mL/kg)		453.0 ± 169.6
Cl (mL/kg/h)	71.9 ± 13.5	73.8 ± 15.7 ^b^
AUC_last_ (µg·h/mL)		139.1 ± 28.6
AUC_0–∞_ (µg·h/mL)	143.4 ± 25.0	140.9 ± 28.5 ^b^
AUMC_last_ (µg·h^2^/mL)		599.4 ± 188.5
AUMC_0–∞_ (µg·h^2^/mL)	636.0 ± 250.2	654.4 ± 192.5
MRT_last_ (h)		4.25 ± 0.63
MRT_0–∞_ (h)	4.38 ± 1.45	4.60 ± 0.69

A, zero-time intercept for the α-phase; B, zero-time intercept for the β-phase; α, distribution rate constant; β, elimination phase rate constant; λ, slope of terminal phase; k_10_, elimination rate constant; k_12_ and k_21_, microrate constants for the drug’s movement between the central and peripheral compartments; t_1/2α_, t_1/2β_, t_1/2λ_, t_1/2k10_, half-lives associated with α, β, λ and k_10_, respectively; V_ss_, volume of distribution at steady-state; V_1_, apparent volume of distribution in the central compartment; V_2_, apparent volume of distribution in the peripheral compartment; V_z_, apparent volume of distribution calculated by the area method; Cl, total body clearance; AUC_last_, area under the plasma concentration-time curve from zero to last collected time point; AUC_0–∞_, area under the plasma concentration-time curve from zero to infinity; AUMC, area under the first moment curve; MRT, mean residence time. ^a^ Significantly different from compartmental parameter (*t* test, *p* ≤ 0.05); ^b^ no significant differences with compartmental parameter.

**Table 2:** Pharmacokinetic parameters (mean ± SD) of menbutone obtained after IM administration (10 mg/kg) to 12 calves.

Parameters	One-Compartmental	Non-Compartmental
λ (h^−1^)		0.16 ± 0.04 ^c,e^
t_1/2λ_ (h)		4.67 ± 1.11 ^c,e^
k_01_ (h^−1^)	1.63 ± 0.44	
k_10_ (h^−1^)	0.18 ± 0.06	
t_1/2k01_ (h)	0.47 ± 0.20	
t_1/2k10_ (h)	4.30 ± 1.28	
C_max_ (µg/mL)	15.1 ± 4.3	17.0 ± 5.0 ^b^
t_max_ (h)	1.66 ± 0.55	1.63 ± 0.23 ^c^
V/F (mL/kg)	535.9 ± 134.4	
V_z_/F (mL/kg)		557.1 ± 136.5 ^c,e^
Cl/F (mL/kg/h)	88.2 ± 12.8	83.0 ± 12.3 ^a,e^
AUC_last_ (µg·h/mL)		119.1 ± 17.5 ^e^
AUC_0–∞_ (µg·h/mL)	115.6 ± 17.7	123.0 ± 18.5 ^a,e^
AUMC_last_ (µg·h^2^/mL)		757.1 ± 178.7 ^e^
AUMC_0–∞_ (µg·h^2^/mL)		881.7 ± 230.9 ^d^
MRT_last_ (h)		6.32 ± 0.94 ^d^
MRT_0–∞_ (h)		7.10 ± 1.18 ^d^
MAT_last_ (h)		2.07 ± 1.11
MAT_0–∞_ (h)		2.50 ± 1.42
F (%)	83.5 ± 22.4	91.0 ± 24.4

λ, slope of terminal phase; k_01_, absorption rate constant; k_10_, elimination rate constant; t_1/2k01_, t_1/2k10_, t_1/2λ_, half-lives associated with k_01_, k_10_ and λ, respectively; C_max_, maximum plasma concentration; t_max_, time to reach C_max_; V_z_/F, apparent volume of distribution; Cl/F, apparent clearance; AUC_last_, area under the plasma concentration-time curve from zero to last collected time point; AUC_0–∞_, area under the plasma concentration-time curve from zero to infinity; AUMC, area under the first moment curve; MRT, mean residence time; MAT, mean absorption time; F, fraction of dose absorbed. ^a^ Significantly different from compartmental parameter (*t* test, *p* ≤ 0.05); ^b^ significantly different from compartmental parameter (Wilcoxon signed-rank test, *p* ≤ 0.05); ^c^ no significant differences with compartmental parameter (for V_z_/F, λ and t_1/2λ_ comparisons were made with V/F, k_10_ and t_1/2k10_, respectively); ^d^ significantly different from IV parameter (*t* test, *p* ≤ 0.05); ^e^ no significant differences with IV parameter (for V_z_/F and Cl/F comparisons were made with V_z_ and Cl, respectively).

## Data Availability

The original contributions presented in the study are included in the article/Supplementary Material; further inquiries can be directed to the corresponding author.

## Supplementary Materials

The following supporting information can be downloaded at https://www.mdpi.com/article/10.3390/ani14172540/s1, Figure S1: Individual plasma concentrations of menbutone obtained after intravenous (**O**) and intramuscular (**▽**) administration (10 mg/kg) to 12 calves; Table S1: Results from linear regression analysis of menbutone calibration curves; Table S2: Individual and mean ± SD plasma concentrations (µg/mL) of menbutone obtained after intravenous administration (10 mg/kg) to 12 calves; Table S3: Individual and mean ± SD plasma concentrations (µg/mL) of menbutone obtained after intramuscular administration (10 mg/kg) to 12 calves.
