# Supplementary material for: Pharmacokinetic Pattern of Menbutone in Calves after Single Intravenous and Intramuscular Administration
Source: Animals (Basel). 2024 Aug 31;14(17):2540. doi: 10.3390/ani14172540 (PMC11393952; doi:10.3390/ani14172540)
Supplement: Supplementary file 1 [file animals-14-02540-s001.zip › animals-3171821-supplementary.pdf]

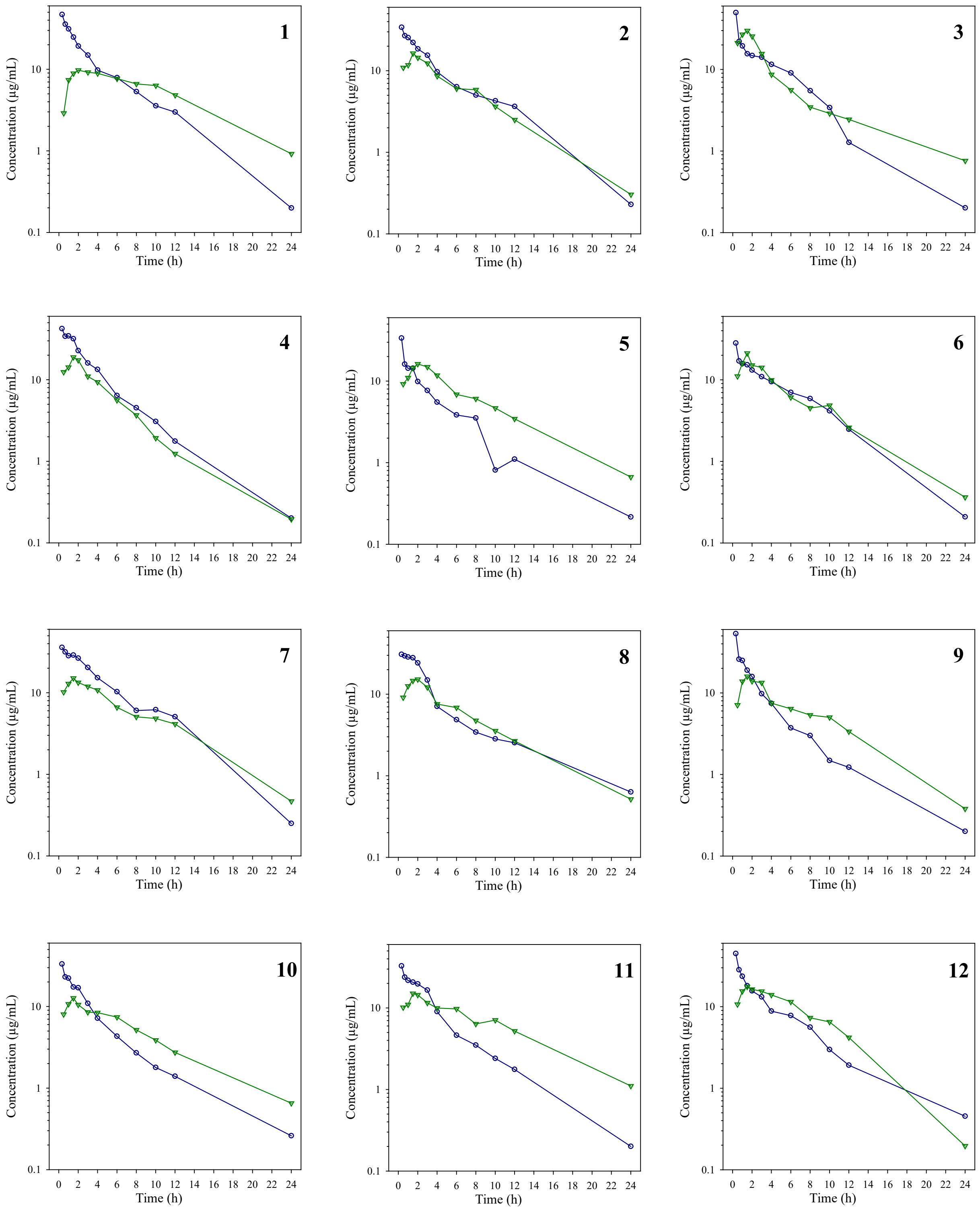

Supplementary Figure S1. Individual plasma concentrations of menbutone obtained after intravenous (○) and intramuscular (▽) administration (10 mg/kg) to 12 calves.

Supplementary Table S1. Results from linear regression analysis of menbutone calibration curves.

**Characteristic**	
Curve 1 (R^2)	Y = 0.3524 X – 0.0083 (R^2 = 0.996)
Curve 2 (R^2)	Y = 0.2867 -0.016 (R^2 = 0.998)
Curve 3 (R^2)	Y = 0.2947 X + 0.0186 (R^2 = 0.996)
LLOQ (µg/mL)	0.2
LOD (µg/mL)	0.09
Recovery (%) (Mean ± SD)	98.34 ± 8.87

SD: standard deviation.

Supplementary Table S2. Individual and mean ± SD plasma concentrations (μg/mL) of menbutone obtained after intravenous administration (10 mg/kg) to 12 calves.

**Time** (h)	**Concentrations** (μg/mL)												**Mean ± SD**
	**1**	**2**	**3**	**4**	**5**	**6**	**7**	**8**	**9**	**10**	**11**	**12**	
0.33	46.785	34.026	49.579	42.224	33.556	28.198	35.923	30.758	53.150	33.114	32.603	44.636	38.713 ± 8.190
0.66	35.656	26.747	21.958	34.031	16.070	17.011	31.655	29.445	25.864	22.982	23.808	28.255	26.123 ± 6.155
1	31.138	25.334	19.323	34.316	14.343	15.754	28.405	28.421	24.943	22.225	21.703	23.513	24.118 ± 5.959
1.5	24.699	22.122	15.508	31.751	14.319	15.247	28.892	27.828	18.967	17.319	20.678	18.029	21.280 ± 5.823
2	19.203	18.480	14.767	22.672	9.849	13.163	26.484	24.095	15.798	16.929	19.605	15.570	18.051 ± 4.742
3	14.865	15.391	14.089	15.982	7.646	10.916	20.356	14.885	9.744	10.905	16.489	13.160	13.702 ± 3.454
4	9.758	9.669	11.496	13.334	5.506	9.470	15.228	7.059	7.432	7.160	8.986	8.801	9.491 ± 2.763
6	7.885	6.331	9.039	6.386	3.839	7.000	10.302	4.853	3.714	4.312	4.610	7.774	6.337 ± 2.139
8	5.318	5.021	5.461	4.524	3.524	5.888	6.042	3.421	2.991	2.700	3.500	5.585	4.498 ± 1.205
10	3.559	4.268	3.394	3.064	0.809	4.162	6.185	2.825	1.482	1.788	2.400	2.972	3.076 ± 1.429
12	2.983	3.631	1.273	1.763	1.102	2.474	5.067	2.533	1.221	1.391	1.761	1.910	2.259 ± 1.174
24	0.200	0.229	0.200	0.200	0.215	0.208	0.250	0.631	0.200	0.260	0.200	0.453	0.270 ± 0.134

SD: standard deviation.

Supplementary Table S3. Individual and mean ± SD plasma concentrations (μg/mL) of menbutone obtained after intramuscular administration (10 mg/kg) to 12 calves.

**Time** (h)	**Concentrations** (μg/mL)												**Mean ± SD**
	**1**	**2**	**3**	**4**	**5**	**6**	**7**	**8**	**9**	**10**	**11**	**12**	
0.5	2.898	10.887	21.126	12.418	9.194	11.053	10.175	9.083	7.082	8.005	10.149	10.648	10.227 ± 4.219
1	7.379	11.697	26.850	14.209	10.985	15.926	12.923	12.557	13.811	10.709	10.963	15.453	13.622 ± 4.775
1.5	8.910	16.343	29.854	18.838	14.489	21.149	15.107	14.609	15.905	12.666	15.001	17.524	16.700 ± 5.139
2	9.743	14.464	25.477	17.360	16.232	15.109	13.360	15.202	13.923	10.513	14.438	16.357	15.181 ± 3.938
3	9.204	12.308	15.575	10.981	14.946	14.159	11.919	12.133	13.364	8.523	11.551	15.404	12.506 ± 2.292
4	8.898	8.587	8.662	9.380	11.771	9.939	10.763	7.576	7.483	8.408	9.945	13.915	9.611 ± 1.843
6	7.678	6.024	5.584	5.615	6.870	6.101	6.612	6.853	6.398	7.404	9.731	11.469	7.195 ± 1.754
8	6.621	5.844	3.445	3.674	6.077	4.519	5.071	4.751	5.354	5.161	6.358	7.293	5.347 ± 1.159
10	6.310	3.637	2.891	1.927	4.669	4.863	4.838	3.542	5.013	3.872	7.103	6.499	4.597 ± 1.530
12	4.821	2.502	2.436	1.238	3.442	2.598	4.147	2.668	3.360	2.731	5.192	4.181	3.276 ± 1.138
24	0.919	0.303	0.759	0.200	0.666	0.364	0.468	0.515	0.380	0.652	1.104	0.200	0.543 ± 0.285

SD: standard deviation.
